# The NRF2 signaling pathway in hepatocellular carcinoma: dual roles, epigenetic reprogramming, and therapeutic opportunities in metabolic vulnerability

**DOI:** 10.3389/fonc.2026.1874956

**Published:** 2026-08-05

**Authors:** Yue Wang, Lingwei Gao, Haobo Yang, Dan Ouyang, Jiale Zhu, Weixiong Jian, Jianzhong Cao

**Affiliations:** 1 College of Traditional Chinese Medicine, Hunan University of Traditional Chinese Medicine, Changsha, China; 2 National Key Discipline of Traditional Chinese Medicine Diagnostics, Hunan Provincial Key Laboratory, Hunan University of Traditional Chinese Medicine, Changsha, China

**Keywords:** epigenetic remodeling, ferroptosis resistance, hepatocellular carcinoma, metabolic reprogramming, metabolic vulnerability, NRF2 signaling, redox homeostasis

## Abstract

Hepatocellular carcinoma (HCC) is a highly heterogeneous malignant tumor with a poor prognosis; its onset and progression are closely associated with persistent oxidative stress and metabolic reprogramming. Nuclear factor E2-related factor 2 (NRF2), as a key transcription factor regulating redox homeostasis, exerts a cytoprotective effect during chronic liver injury by inducing the expression of antioxidant and detoxification genes; however, following tumor formation, it may undergo abnormal, sustained activation, thereby contributing to metabolic adaptation and treatment resistance in HCC. Based on evidence from existing basic and translational research, this review systematically integrates the multilevel regulatory networks of NRF2 in HCC, including the classical KEAP1–NRF2 ubiquitination and degradation pathway, the p62-mediated non-classical activation mechanism, and the cross-regulation of metabolic stress signaling pathways such as AMPK–mTOR. Based on this, we summarize the stage-dependent dual role of NRF2 in HCC progression: in early-stage chronic liver injury, it primarily limits oxidative stress and DNA damage; whereas in advanced tumors, it enhances tumor cell survival and promotes invasion, metastasis, and multidrug resistance through metabolic reprogramming, such as by promoting glucose metabolism, glutathione synthesis, and the pentose phosphate pathway. Furthermore, NRF2 enhances resistance to ferrocytosis by reshaping the GSH–GPX4 axis and the iron homeostasis network, and may participate in the regulation of the tumor immune microenvironment, thereby collectively shaping the treatment-resistant characteristics of HCC. Based on these context-dependent changes, NRF2-related metabolic pathways and redox defense systems exhibit potential metabolically targetable vulnerabilities, providing a theoretical basis for combined induction of ferrocytosis and metabolic intervention. Overall, NRF2 exhibits significant context-dependence and dual biological effects in HCC; its precise regulatory mechanisms and clinical translational value remain to be further validated.

## 1 Introduction

Hepatocellular carcinoma (HCC) is one of the leading causes of cancer incidence and mortality worldwide. Hepatocellular carcinoma (HCC) is one of the malignant tumors with high incidence and mortality rates worldwide. According to the report “Global Cancer Statistics 2022”, there were approximately 865,269 new cases of liver cancer and 757,948 deaths worldwide in 2022, making it the third leading cause of cancer-related deaths (1). Because liver cancer typically has an insidious onset and a poor prognosis, its disease burden has become a major challenge in global public health. Notably, the global burden of liver cancer is projected to continue to increase in the coming decades. A predictive model developed by Harriet Rumgay et al. based on the GLOBOCAN 2020 database indicates that, driven by factors such as population growth and aging, the annual number of new liver cancer cases worldwide is projected to increase by 48.3% by 2040, reaching approximately 1.28 million; during the same period, the number of deaths is projected to rise by 50.1%, reaching approximately 1.14 million (2). Liver cancer has become one of the leading causes of cancer-related deaths in many countries and regions, and its incidence and mortality burden continue to rise. Therefore, elucidating the molecular mechanisms underlying the development of liver cancer and exploring effective prevention and treatment strategies are of great significance for reducing the global burden of liver cancer.

The multistep pathogenesis of liver cancer is widely recognized as an inflammation-driven carcinogenic process closely associated with unique environmental and viral etiologies, including chronic hepatitis B virus (HBV) or hepatitis C virus (HCV) infection, chronic alcohol abuse, and the increasing prevalence of metabolic dysfunction-associated steatohepatitis (MASH/NASH) (3–5). Although these etiologies have different initiating mechanisms, they all induce chronic inflammation, mitochondrial dysfunction, lipid peroxidation, and abnormal accumulation of reactive oxygen species (ROS) through persistent liver damage, thereby creating a persistent oxidative stress microenvironment. Long-term oxidative stress not only promotes genomic instability and epigenetic abnormalities in hepatocytes but also drives the remodeling of the liver microenvironment, creating favorable conditions for the development and progression of hepatocellular carcinoma (6).

Nuclear factor E2-related factor 2 (NRF2) is a key transcription factor that maintains cellular redox homeostasis; under physiological conditions or during acute stress, it exerts a cytoprotective effect by inducing the expression of antioxidant and detoxification-related genes (7, 8). However, during the long-term progression of chronic liver diseases such as HBV/HCV infection, alcoholic liver disease, and MASH, persistent inflammation and oxidative stress can lead to abnormal, sustained activation of NRF2 (3). In this context, in addition to maintaining antioxidant defense, NRF2 can also enhance tumor cells’ metabolic adaptability, ability to maintain redox homeostasis, and stress tolerance, thereby promoting tumor survival and progression (9, 10). A growing body of research suggests that the role of NRF2 in tumors is highly context-dependent, and its biological effects are jointly influenced by factors such as disease stage, cell type, and microenvironmental conditions (11–13).

Authoritative genomic studies from large-scale cohorts such as the The Cancer Genome Atlas (TCGA) and the International Cancer Genome Consortium (ICGC)—including a high-depth sequencing matrix of 363 primary HCC samples—have shown that human liver cancer tissues are commonly associated with high-frequency somatic mutations in classic tumor suppressor or differentiation genes such as *TP53* (31%) and *CTNNB1* (27%). However, it is crucial to note that *NFE2L2* (encoding NRF2) and its endogenous inhibitory protein KEAP1—which serve as central transcriptional hubs for intracellular antioxidant defense and redox homeostasis—exhibit direct mutation rates of only approximately 3% and 5%, respectively, in human HCC cohorts. Although these mutations are highly concentrated within the conserved DLG and ETGE key motifs in the NRF2 Neh2 domain, they can lead to intrinsic hyperactivation of NRF2 by impairing KEAP1’s substrate-binding capacity (12, 14–16). However, unlike mutations in the *NFE2L2* or *KEAP1* genes commonly found in tumors such as non-small cell lung cancer, abnormal NRF2 activation in HCC does not rely entirely on genetic alterations. Existing studies indicate that non-classical mechanisms—such as p62/SQSTM1 accumulation caused by autophagy defects, epigenetic dysregulation, and metabolic stress—can all promote sustained NRF2 activation and contribute to the onset, progression, and treatment resistance of liver cancer (17). It is worth noting that while the p62-KEAP1-NRF2 axis has been relatively well validated in HCC models, some epigenetic and metabolic regulatory mechanisms are still primarily derived from studies of other solid tumors, and their roles in HCC require further confirmation (10, 18).

**Figure 1:**
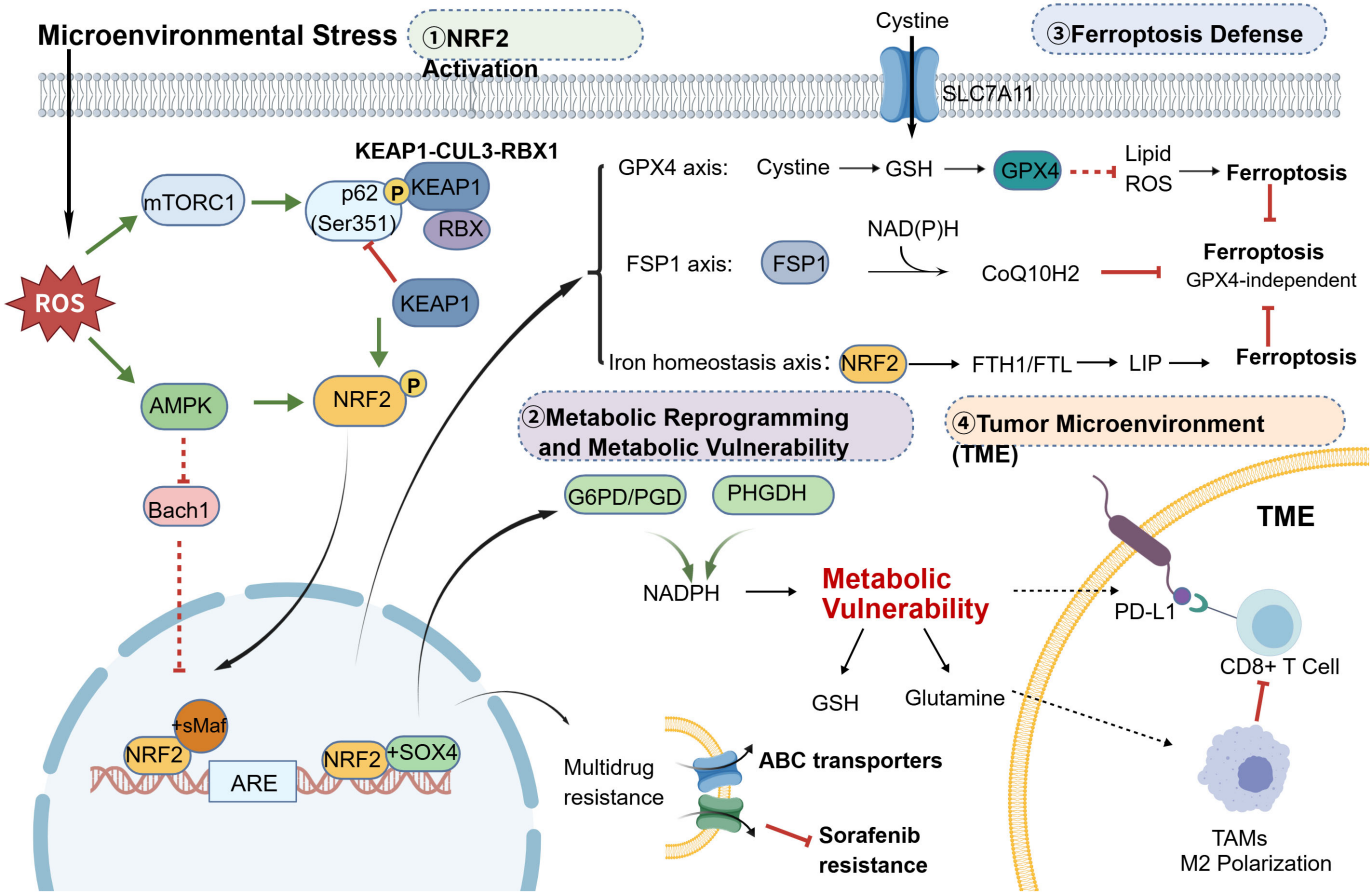
Mechanisms of NRF2 activation and its multifaceted roles in ferroptosis resistance, metabolic reprogramming, drug resistance, and tumor microenvironment remodeling. Schematic overview of NRF2-mediated metabolic adaptation in hepatocellular carcinoma. Microenvironmental stress activates NRF2 through canonical and non-canonical pathways, leading to antioxidant responses, metabolic reprogramming, ferroptosis resistance, and multidrug resistance. These adaptations create metabolic vulnerabilities while promoting immune evasion through tumor microenvironment remodeling. ABC Transporters, ATP-Binding Cassette Transporters; AMPK, 5'- AMP-Activated Protein Kinase; ARE, Antioxidant Response Element; Bach1, BTB Domain and CNC Homolog 1; CUL3, Cullin 3; CoQ10 / CoQ10H2, Coenzyme Q10 / Reduced Coenzyme Q10; FSP1, Ferroptosis Suppressor Protein 1; FTH1 / FTL, Ferritin Heavy Chain 1 / Ferritin Light Chain; G6PD / PGD, Glucose-6-Phosphate Dehydrogenase / 6-Phosphogluconate Dehydrogenase; GLS, Glutaminase; GPX4, Glutathione Peroxidase 4; GSH, Reduced Glutathione; KEAP1, Kelch-like ECH-associated protein 1; LIP, Labile Iron Pool; MCM4, Minichromosome Maintenance Complex Component 4; mTORC1, Mechanistic Target of Rapamycin Complex 1; NADPH, Nicotinamide Adenine Dinucleotide Phosphate; NAT10, N-Acetyltransferase 10; NRF2, Nuclear Factor Erythroid 2-Related Factor 2; PD-L1, Programmed Death-Ligand 1; PHGDH, Phosphoglycerate Dehydrogenase; p62 (SQSTM1), Sequestosome 1; RBX1, RING-Box Protein 1; ROS, Reactive Oxygen Species; SLC7A11, Solute Carrier Family 7 Member 11; sMaf, Small Musculoaponeurotic Fibrosarcoma Protein; SOX4, SRY-Box Transcription Factor 4; TAMs, Tumor-Associated Macrophages.

Although the NRF2 signaling pathway has been extensively studied, existing reviews have largely focused on its classical KEAP1–NRF2 regulatory model and antioxidant functions. There remains a relative lack of systematic integration regarding the synergistic regulation among different metabolic pathways and their complex interactions with ferroptosis, epigenetic regulation, the tumor immune microenvironment, and treatment resistance (19–21). A growing body of research indicates that while tumor cells gain a metabolic advantage, they also gradually develop a high dependence on key metabolic enzymes, nutrient supply, redox homeostasis, and anti-ferroptosis systems; these dependencies provide new opportunities for intervention through metabolism-targeted therapies and combination therapies. Currently, there are significant variations in the sources and strength of relevant evidence: while some mechanisms have been validated in HCC models, other conclusions are primarily derived from studies on other solid tumors and require further confirmation in HCC (22–24). Therefore, as a narrative review, this article aims to, from an HCC-specific perspective, highlight the role of metabolic reprogramming in tumor progression, adaptation to oxidative stress, immune regulation, and treatment resistance. Furthermore, it objectively evaluates the existing evidence and its limitations across three dimensions—metabolic advantage, metabolic vulnerability, and potential intervention strategies—and explores the potential value and challenges of targeting NRF2-related metabolic and redox adaptation mechanisms in the precision treatment of hepatocellular carcinoma (**Figure 1**).

## 2 Classical and non-classical regulatory mechanisms of NRF2

### 2.1 The classic KEAP1-NRF2 ubiquitination and degradation pathway

Under physiological conditions, the steady-state levels and subcellular localization of the core transcriptional factor NRF2 are subject to strict and precise post-transcriptional regulation by the ubiquitin-proteasome system (UPS) (25). The central negative regulator of this repressive mechanism is the Kelch-like ECH-associated protein 1 (KEAP1), which was first cloned and named by the team led by Masayuki Yamamoto; it exerts its transcriptional repressive function by specifically binding to the evolutionarily conserved N-terminal Neh2 regulatory domain of NRF2 (26). Structurally, KEAP1 belongs to the BTB-Kelch family and utilizes its N-terminal BTB domain and the adjacent “3-box” motif to mediate its own homodimerization and recruit the Cullin 3 (CUL3) scaffold to assemble with RBX1 into a functional KEAP1–CUL3–RBX1 E3 ubiquitin ligase complex (27–29). In recent years, some researchers have proposed that the KEAP1–NRF2 pathway is better understood as a dynamic redox homeostasis regulatory system (redoxostat) rather than a simple “on-off” antioxidant pathway; however, this concept remains primarily based on theoretical models and systems biology analyses and requires further experimental validation (30).

In terms of spatial topology, based on high-resolution crystal structure analysis, the KEAP1 adapter dimer exhibits a dual-site recognition pattern for NRF2 substrates through its C-terminal Kelch domains, known as the “hinge–latch” model (12, 31–33). The two Kelch domains specifically recognize two cis-degradons within the NRF2 Neh2 domain: the high-affinity ETGE motif (which acts as a fixed “hinge”) and the low-affinity DLG motif (which acts as a locking “latch”). This dual-site recognition recruits and stretches the Neh2 domain of NRF2 to the catalytic center of the ligase complex, forcing its lysine (Lys)-rich region to become fully exposed to undergo polyubiquitination and rapid degradation in the cytoplasm, thereby limiting the expression of antioxidant-related genes to low levels (27–29, 33, 34). Through label-free quantitative interactomics analysis, the CUL3-KEAP1 complex may be involved in the ubiquitination regulation of prolyl hydroxylase domain-containing protein 2 (PHD2), thereby establishing a potential link between oxidative stress and the hypoxic response; however, its physiological significance in HCC remains to be further investigated (28).

When hepatocellular carcinoma cells are exposed to a state of persistent oxidative stress, the NRF2 pathway is often activated (6). Mechanistically, this process is associated with the oxidation or electrophilic modification of key cysteine residues in KEAP1 (such as Cys151, Cys273, and Cys288), which inhibits KEAP1-CUL3-mediated ubiquitination and degradation of NRF2 (27, 34, 35). High-resolution structural biology studies further indicate that electrophile-induced classical NRF2 activation is primarily achieved by inhibiting the KEAP1-CUL3 complex-mediated ubiquitination process, rather than simply causing dissociation of the DLG lock site (32). As the ubiquitination and degradation pathway is inhibited, newly synthesized NRF2 escapes UPS-mediated clearance and rapidly accumulates, subsequently translocating into the cell nucleus. Within the nucleus, the bZIP domain of NRF2 forms a heterodimeric complex with the small Maf (sMaf) protein, which specifically binds to the antioxidant response element (ARE) in the promoter regions of target genes, thereby initiating cellular stress adaptation programs. Following nuclear translocation, NRF2 induces the expression of various antioxidant and stress-adaptation-related genes, including HMOX1, NQO1, GCLC, SLC7A11, and GPX4. Among these, SLC7A11 and GPX4 are involved in glutathione metabolism and the ferroptosis defense network; these pathways play a crucial role in HCC cells’ adaptation to sustained oxidative stress, maintenance of metabolic homeostasis, and resistance to ferroptosis (36, 37).

**Figure 2:**
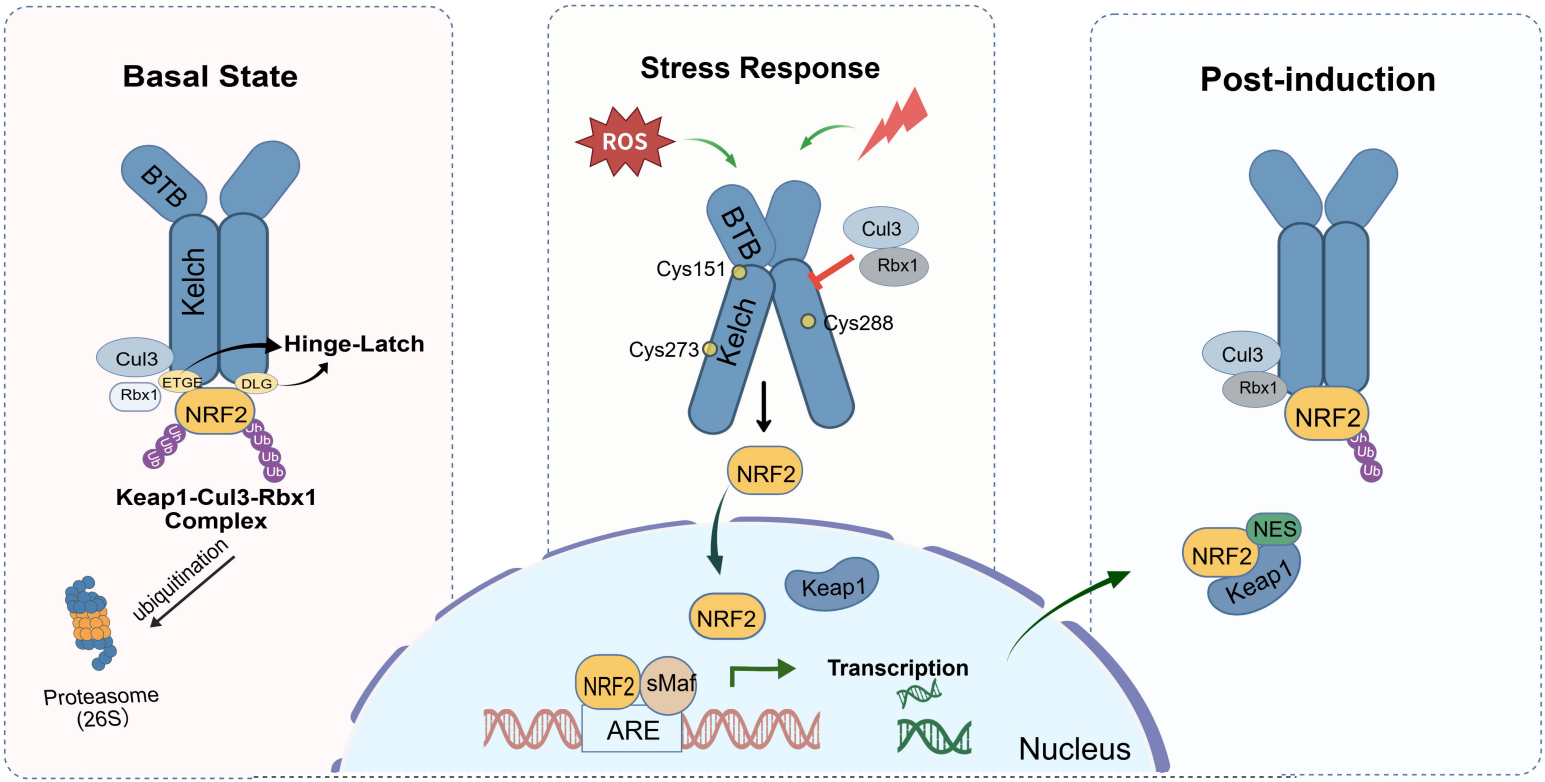
Canonical activation and regulation of the KEAP1–NRF2 signaling pathway. Regulation of NRF2 activation under basal, stress, and post-induction conditions. Under basal conditions, NRF2 is sequestered by the KEAP1–Cullin3 (Cul3) -Rbx1 E3 ubiquitin ligase complex through the ETGE and DLG motifs in a “hinge–latch” manner, leading to its continuous ubiquitination and proteasomal degradation. Upon oxidative stress, reactive oxygen species (ROS) modify critical cysteine residues of KEAP1 (Cys151, Cys273, and Cys288), disrupting the KEAP1– NRF2 interaction and inhibiting ubiquitination. Stabilized NRF2 accumulates and translocates into the nucleus, where it binds to antioxidant response elements (ARE) and activates transcription of target genes. In the post-induction phase, NRF2 is exported from the nucleus via interaction with KEAP1 containing a nuclear export signal (NES), leading to reformation of the degradation complex and restoration of basal homeostasis. ARE, antioxidant response element; Cul3, Cullin3; KEAP1, Kelch-like ECH- associated protein 1; NES, nuclear export signal; NRF2, nuclear factor erythroid 2-related factor 2; Rbx1, RING-box protein 1; ROS, reactive oxygen species.

During the post-induction phase, as the intracellular redox balance gradually recovers, Keap1 actively enters the nucleus and binds to Nrf2, causing it to dissociate from the ARE site. Subsequently, the Nrf2–Keap1 complex is transported back to the cytoplasm via its nuclear export sequence (NES), thereby restoring the signaling pathway to its basal state (19, 38, 39). Studies have shown that blocking the NES domain of KEAP1 leads to abnormal accumulation of the NRF2–KEAP1 complex in the nucleus, thereby prolonging ARE-dependent transcriptional activity and hindering the timely termination of the signaling pathway (27, 38). Recent research suggests that the degradation of ubiquitinated NRF2 may require the involvement of the ATP-dependent disassembly enzyme p97/VCP. Shakya et al. found that p97 promotes the dissociation of ubiquitinated NRF2 from the KEAP1 complex and its subsequent delivery to the proteasome for degradation, thereby participating in the regulation of NRF2 protein homeostasis. Based on these findings, the authors proposed the concept of an “NRF2-p97-NRF2 negative feedback loop” to explain the dynamic regulatory mechanisms involved in signal termination during the late stages of NRF2 activation. However, this model is currently based primarily on studies at the cellular and molecular levels, and its physiological significance in different tissues and within the pathological context of HCC requires further validation (40). It should be noted that the aforementioned classical KEAP1–NRF2 regulatory mechanism has been thoroughly validated in basic biological research and represents the most well-established model of NRF2 activation in HCC to date. Nevertheless, systematic comparative studies on the degree of activation, dynamic changes, and relative contribution of this pathway to tumor progression across different etiological backgrounds (HBV, HCV, MASH, and alcoholic liver disease) are still lacking. Therefore, future research should further elucidate the dynamic regulatory patterns of the classical KEAP1–NRF2 pathway in the development and progression of HCC by integrating different etiological backgrounds and clinical cohorts (**Figure 2**).

### 2.2 Non-canonical regulation

#### 2.2.1 Competitive binding of p62 and KEAP1

In addition to classic electrophilic redox signaling, the activity of the NRF2 signaling pathway is also regulated by p62 (also known as SQSTM1), a core molecule in the autophagy regulatory network. As a multifunctional chaperone protein and selective autophagy receptor, p62 serves as a central molecular hub linking the autophagy-lysosomal pathway with the NRF2 signaling pathway (41). At the molecular level, p62 binds to the Kelch domain of KEAP1 via its KEAP1 interaction region (KIR), thereby disrupting KEAP1-mediated negative regulation of NRF2 and promoting activation of the NRF2 signaling pathway (42, 43). This non-canonical activation process is gated by tiered post-translational modifications. Against the backdrop of intracellular accumulation of ubiquitinated protein aggregates, p62 is first phosphorylated at Ser403 in its ubiquitin-binding domain (UBA) by upstream kinases (such as TBK1 or CK2) to enhance its recognition and capture of ubiquitinated substrates (44); simultaneously, the serine ubiquitination of SQSTM1 itself also contributes to the regulation of intracellular redox homeostasis (45). Subsequently, metabolic stress activates the mammalian target of rapamycin complex 1 (mTORC1) pathway, which directly mediates a key phosphorylation modification at Ser351 within the KIR domain of p62. Phosphorylation of p62 at this site significantly increases its binding affinity for KEAP1, leading to the sequestration of KEAP1 into autophagosome aggregates (17, 42). This competitive binding reduces KEAP1’s ability to target NRF2 for degradation, allowing newly synthesized NRF2 to translocate to the nucleus and activate ARE-driven expression of downstream target genes (17). Furthermore, the p62-KEAP1 complex, along with recruited ubiquitinated substrates, is encapsulated into autophagosomes and ultimately degraded via the autophagy-lysosomal pathway, thereby clearing intracellular protein aggregates while activating downstream antioxidant responses (43, 46, 47). Once this interface is disrupted due to mutations at specific sites in the *p62* gene, it leads to the obstruction of selective autophagy and disrupts the intracellular antioxidant defense response (44). In pathological samples of primary hepatocellular carcinoma (HCC), the association between abnormal p62 accumulation and sustained NRF2 signaling has been reported in multiple studies. Studies have shown that NRF2 promotes the expression of *SQSTM1* (*p62*) at the transcriptional level, and the accumulated p62 protein further binds to and sequesters KEAP1, thereby forming a positive feedback loop that promotes sustained NRF2 activation (17, 48). This mechanism is considered one of the key pathways for non-canonical NRF2 activation in HCC. In recent years, several pharmacological studies have suggested that the p62-KEAP1-NRF2 axis may be involved in the regulation of oxidative stress and ferroptosis in HCC cells. For example, combination therapy with metformin and sorafenib, as well as the natural product curcumin, have been reported to modulate the p62–KEAP1–NRF2 signaling axis, accompanied by increased levels of lipid peroxidation and the emergence of ferroptosis-related phenotypes (46, 47). However, these findings currently stem primarily from cellular and animal experiments, and their clinical translational value remains to be further validated. Additionally, Feng et al. (49) reported a potential upstream regulatory mechanism: the oncogene RPRD1A competes with TRIM21 for binding to p62, thereby reducing p62 degradation and maintaining NRF2 in an activated state, which in turn affects the sensitivity of HCC cells to cisplatin treatment. However, this mechanism is currently based primarily on the results of a single study, and its generalizability across different HCC models and clinical cohorts requires further validation. Overall, the sustained activation of the p62-KEAP1-NRF2 axis in HCC has been well supported by mechanistic studies; however, some upstream regulatory factors and related therapeutic strategies remain in the exploratory stage, and their clinical significance requires further research evidence to confirm.

#### 2.2.2 Interactions between AMPK-mTOR and NRF2

5’-AMP-activated protein kinase (AMPK) is a key energy sensor in cells. It is activated under conditions such as nutrient deprivation, oxidative stress, and mitochondrial dysfunction, and is involved in regulating cellular redox homeostasis and metabolic adaptation processes (50, 51). Recent studies have shown that AMPK and NRF2 share multifaceted functional interactions and jointly participate in the adaptive regulation of cells in response to stressful environments. Mechanistic studies have shown that AMPK can directly regulate NRF2 activity. Joo et al. (52) found that under energy stress conditions, AMPK phosphorylates Ser550 on mouse Nrf2 (corresponding to Ser558 on human NRF2), thereby inhibiting its nuclear export and promoting its accumulation in the nucleus, which enhances the transcriptional activity of certain NRF2 target genes. Subsequently, Matzinger et al. (53) further confirmed through phosphoproteomics and *in vitro* kinase assays that AMPK can directly induce phosphorylation at multiple serine sites (including Ser374, Ser408, and Ser433) on human NRF2, thereby modulating the transcriptional output profile of specific NRF2 target genes rather than simply enhancing the expression of all NRF2 downstream genes. Nevertheless, current evidence primarily stems from cellular and animal experiments, and the biological significance and clinical relevance of this regulatory mechanism in HCC tissue remain to be further validated.

In addition to direct phosphorylation, AMPK can also indirectly influence NRF2 activity by regulating other signaling nodes. Studies have shown that AMPK activation can inhibit glycogen synthase kinase 3β (GSK3β) activity, thereby reducing NRF2 degradation and enhancing its stability (52, 54). At the same time, AMPK can also enhance the expression of certain NRF2 target genes by negatively regulating the transcriptional repressor Bach1 (54). It is worth noting that most of these studies were conducted in non-HCC models, and their specific roles and clinical significance in hepatocellular carcinoma remain to be further validated. In addition to the direct and indirect regulatory mechanisms mentioned above, a growing body of research suggests that autophagy may be involved in the functional link between AMPK and NRF2 signaling. As a selective autophagy receptor, p62/SQSTM1 promotes NRF2 activation by binding to KEAP1 and attenuating its negative regulation of NRF2; abnormal accumulation of p62 has been shown to be closely associated with the development and progression of HCC (55, 56). Particularly in HCV-associated HCC, p62-dependent sustained activation of NRF2 promotes tumor cell survival and malignant progression (55). However, there is currently a lack of direct evidence demonstrating that AMPK can drive sustained NRF2 activation through a p62-dependent KEAP1 degradation mechanism; therefore, the specific regulatory mechanism of the AMPK–p62–NRF2 axis in HCC remains to be further elucidated.

Notably, AMPK, NRF2, and the unfolded protein response (UPR) are all involved in the cell’s adaptation to stressful environments (57–59). Previous studies have shown that abnormal activation of the UPR is closely associated with HCC proliferation, metabolic reprogramming, invasion and metastasis, and poor prognosis (57, 58). On the other hand, a functional regulatory relationship exists between AMPK and NRF2, and experimental studies suggest that AMPK activation enhances NRF2/HO-1 signaling and interacts with the UPR pathway (60). Furthermore, autophagy dysregulation and abnormalities in protein homeostasis are considered key features of HCC development, and there are extensive links between autophagy-related networks and NRF2 signaling (56). However, direct evidence supporting the formation of a unified regulatory network involving these pathways in HCC remains relatively limited, and the key molecular connection points and their biological significance still require further elucidation.

## 3 The context-dependent dual role of NRF2 in the progression of liver cancer

NRF2 is a key transcription factor encoded by the *NFE2L2* gene that plays a crucial role in maintaining cellular redox homeostasis, as well as in antioxidant and anti-inflammatory responses (61–63). Satoh et al. recent study has shown that NRF2 exhibits a “double-edged sword” effect during tumorigenesis and tumor progression. In normal tissues or during the early stages of tumor development, NRF2 exerts a protective role by activating the antioxidant defense system; however, following tumor formation, abnormal activation of NRF2 may confer survival and evolutionary advantages to tumor cells (64).

### 3.1 A cellular protective barrier in early chronic liver damage

During the initial and precancerous stages of liver cancer development, NRF2 plays an indispensable role as a cellular protective “genomic gatekeeper” (65). In the early stages of liver cancer development—including against the backdrop of chronic liver diseases such as chronic viral hepatitis, MASH, and liver fibrosis—NRF2 primarily exerts a protective effect by maintaining liver homeostasis and limiting tumor initiation (65–68). As a central transcription factor in the cellular response to oxidative stress, NRF2 binds to the antioxidant response element (ARE) to induce the expression of antioxidant and detoxification-related genes such as NQO1, GCLC, GCLM, HMOX1, and other antioxidant and detoxification-related genes, thereby promoting the scavenging of reactive oxygen species (ROS), enhancing glutathione synthesis, and maintaining cellular redox balance (65, 68). Studies using chemical carcinogenesis models have shown that exposure to diethylnitrosamine (DEN) can induce significant oxidative stress, inflammatory responses, and abnormal hepatocyte proliferation in the early stages of liver carcinogenesis, thereby creating favorable conditions for subsequent tumor development (56). Combined with genetic studies, it was found that Nrf2-deficient mice exhibited more severe DNA damage and a higher incidence of liver tumors following exposure to heterocyclic amine chemocarcinogens, suggesting that NRF2 plays an important protective role in the early stages of liver cancer development (67). On the other hand, NRF2 activation can alleviate toxicant-induced liver damage, inflammatory responses, and fibrosis, thereby reducing the risk of chronic liver disease progressing to hepatocellular carcinoma (66, 68, 69). It is worth noting that this protective effect primarily occurs prior to malignant transformation. In the context of persistent chronic liver injury, the long-term activation of the NRF2 signaling pathway may gradually be co-opted by tumor cells, thereby transforming from a defense mechanism that maintains tissue homeostasis into a factor that promotes tumor adaptation and survival (69–71). Therefore, NRF2 exhibits distinct stage-dependent characteristics in the development of hepatocellular carcinoma: it primarily functions as a tumor suppressor during the tumor initiation phase, but may transform into a key driver promoting tumor progression once the tumor has formed (72, 73).

### 3.2 Progression of advanced liver cancer: metabolic adaptation and drug resistance

However, as HCC enters the stage of malignant progression, the biological role of NRF2 gradually expands from maintaining redox homeostasis to participating in the regulation of tumor.

This is a provisional file, not the final typeset article metabolism. A growing body of research indicates that persistently activated NRF2 is closely associated with the reprogramming of glucose metabolism, amino acid metabolism, and redox metabolism, helping HCC cells adapt to oxidative stress, nutritional deprivation, and therapeutic stress (55, 67, 72–75). Notably, these metabolic changes not only enhance the tumor cells’ ability to adapt to their environment but may also increase their metabolic dependency on certain key metabolic processes (such as glutathione metabolism, NADPH production, and serine biosynthesis).

Therefore, NRF2-associated metabolic reprogramming may provide metabolic support for malignant phenotypes such as invasion and metastasis, treatment resistance, and ferroptosis tolerance, while also suggesting that these metabolic networks may harbor potential metabolic vulnerabilities that could be exploited for therapeutic purposes.

#### 3.2.1 Metabolic reprogramming

During the progression of HCC, persistently activated NRF2 gradually evolves from a classical antioxidant regulator into a key regulator of metabolic reprogramming. By promoting metabolic processes such as the nucleotide synthesis and NADPH production, it enhances tumor cells’ adaptability to stressful environments and meets their biosynthetic needs (55, 76). These metabolic reprogramming events increase the dependence of tumor cells on specific metabolic pathways, providing a theoretical basis for therapeutic strategies targeting NRF2-related metabolic vulnerabilities (55, 76).

Xi et al. (76) reported that NRF2 deficiency reduces acetyl-CoA levels and histone acetylation and downregulates the expression of metabolism-related genes such as NQO1, ACO2, and SUCLG2, suggesting that NRF2 maintains metabolic processes in hepatocellular carcinoma cells through a metabolic–epigenetic coupling. At the transcriptional regulation level, enhancer networks and chromatin topological remodeling further amplify NRF2 signaling output. For example, super-enhancer (SE)-mediated enhancer–promoter (EP) looping activates TRIB2 expression, and TRIB2, by competitively binding to KEAP1, disrupts the KEAP1–NRF2 complex, thereby enhancing NRF2 stabilization and nuclear translocation, which continuously drives antioxidant and metabolic adaptation programs (77–79). Furthermore, recent studies have found that SOX4 can form a transcriptional complex with NRF2, jointly promoting PSPH expression and enhancing serine biosynthesis; in NRF2-high/SOX4-high HCC tissues, downstream reprogrammed metabolites such as NADPH, NADH, and succinate are significantly elevated; whereas under oxidative stress conditions, the deletion or knockdown of SOX4 significantly attenuates NRF2’s transcriptional activation of downstream ARE target genes (such as GPX4, GCLM, PGD, and PSPH) (80). These mechanisms collectively promote NRF2-driven metabolic reprogramming, causing tumor cells to gradually develop a sustained dependence on core metabolic networks—including NADPH production, serine metabolism, and redox homeostasis—thereby providing a common metabolic foundation for subsequent EMT, maintenance of tumor stemness, and treatment resistance.

#### 3.2.2 EMT and adaptation to invasion and metastasis

Building on its role in metabolic regulation, NRF2 further promotes the acquisition of enhanced invasion and metastasis capabilities in HCC cells. Studies have shown that NRF2 promotes epithelial–mesenchymal transition (EMT) and metastasis in HCC by regulating key factors such as Snail and MMP2/9 (81, 82). Unlike the inhibitory role of its family member Nrf1, NRF2 tends to lift specific miRNA-mediated suppression and enhance the expression of invasion-related genes (82). Metabolic and redox signaling pathways (such as the ROS–NRF2–Notch1 axis) further amplify its pro-metastatic effects (83). In sorafenib-resistant Huh7 cells, NRF2 knockdown significantly reduced the expression of cancer stem cell markers EPCAM, CD44, and Nanog, while simultaneously decreasing the cells’ migratory and invasive capabilities (74). These studies suggest that NRF2-mediated metabolic reprogramming is closely associated with EMT, invasion, and stemness-related phenotypes. The sustained maintenance of NADPH production, glutathione metabolism, and redox homeostasis helps HCC cells adapt to the persistent oxidative stress environment during invasion and metastasis. A growing body of research suggests that NRF2-related metabolic regulation is closely associated with EMT, tumor stemness, and invasive capacity; these processes may further increase tumor cells’ dependence on relevant metabolic networks and occur in conjunction with malignant phenotypes such as treatment resistance.

#### 3.2.3 Metabolic adaptation and therapeutic resistance

Persistently activated NRF2 is considered a key molecular basis for the development of therapeutic resistance in HCC. A growing body of research indicates that NRF2-mediated resistance is not limited to a single drug but rather enhances the tolerance of HCC cells to chemotherapy, molecularly targeted therapy, and local therapy through a combination of mechanisms: reshaping redox homeostasis, enhancing glutathione (GSH) metabolism and NADPH supply, promoting drug detoxification and efflux, and increasing the cells’ ability to withstand oxidative stress and ferroptosis (72, 73, 84).

In chemotherapy and molecularly targeted therapy, NRF2-driven metabolic adaptation further promotes treatment resistance. On the one hand, NRF2 enhances the detoxification capacity of tumor cells by boosting glutathione metabolism and maintaining redox homeostasis; on the other hand, NRF2 regulates the expression of ATP-binding cassette (ABC) transporters, promoting drug efflux and reducing intracellular drug accumulation. Studies have shown that NRF2 can regulate the expression of ABC transporters, such as ABCG2, thereby conferring a multidrug resistance (MDR) phenotype on tumor cells (73, 74). Furthermore, inhibiting NRF2 activity reduces intracellular glutathione levels and drug efflux capacity in BEL-7402/5-FU-resistant cells, restoring their sensitivity to 5-FU, which further illustrates that NRF2-mediated metabolic adaptation and drug efflux jointly contribute to the development of chemotherapy resistance (85).

In a sorafenib-resistant model, sustained NRF2 activation is not only accompanied by increased expression of ABC transporters but is also closely associated with the phenotype of tumor-initiating cells (TICs). Studies have shown that in sorafenib-resistant Huh7 cells, NRF2 activation is accompanied by increased expression of TIC-associated markers such as EPCAM, CD44, and Nanog; knocking down NRF2 significantly reduces cell migration, invasion, and drug resistance, suggesting that NRF2 maintains the drug-resistant phenotype by coordinating drug efflux, tumor-initiating cell characteristics, and metabolic adaptation (74, 86). Furthermore, FNDC5 can promote the acquisition of sorafenib resistance in HCC cells by activating the PI3K/AKT/NRF2 signaling pathway, whereas inhibition of NRF2 restores cellular sensitivity to sorafenib (87).

In addition to drug efflux, NRF2-mediated antioxidant metabolic remodeling is also a key mechanism underlying treatment resistance. Studies have shown that camptothecin, by inhibiting NRF2, significantly enhances sorafenib-induced lipid peroxidation and ferroptosis, thereby increasing the sensitivity of tumor cells to treatment (88). Clinical specimens from TAE/TACE treatments further demonstrate that NRF2 and its target genes ABCG2 and HMOX1 are persistently highly expressed and are closely associated with the enrichment of tumor-initiating cell markers and poor patient prognosis (89). Furthermore, valproic acid can enhance the sensitivity of HCC cells to proton radiotherapy by inhibiting NRF2 signaling, further suggesting that NRF2 is an important potential intervention target for improving the efficacy of radiotherapy (41).

**Figure 3:**
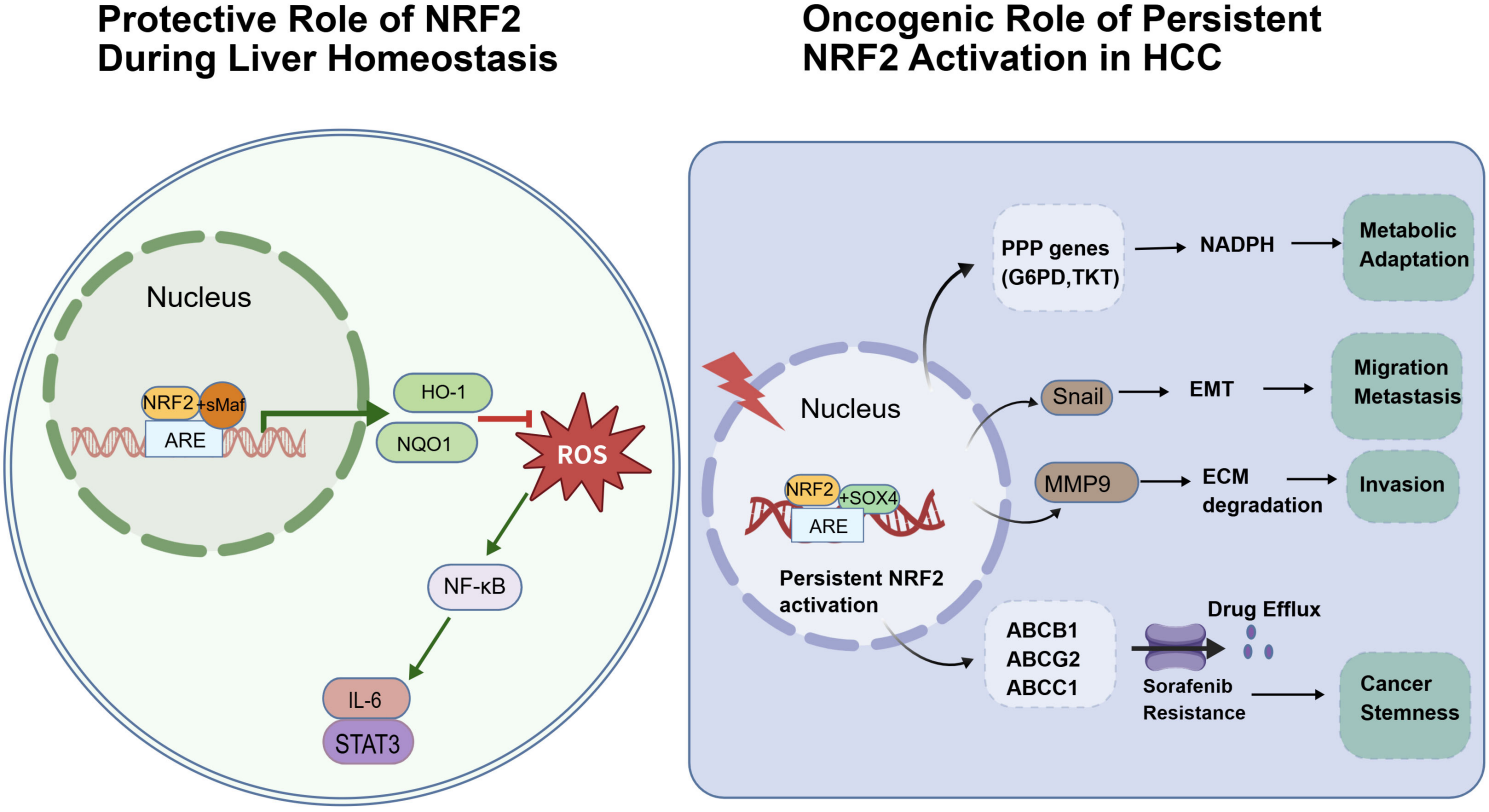
Dual role of NRF2 in hepatocarcinogenesis: protective effects during tumor initiation and pro-tumorigenic functions during tumor progression. In healthy hepatocytes and early tumor initiation, NRF2 activation induces antioxidant response element (ARE)-driven transcription of cytoprotective genes such as NQO1 and HO-1, thereby reducing intracellular reactive oxygen species (ROS) levels and suppressing pro-inflammatory signaling pathways, including NF-κB/IL-6/STAT3. This antioxidant and anti-inflammatory activity contributes to the maintenance of redox homeostasis and limits malignant transformation. In contrast, during liver cancer progression, persistent NRF2 activation promotes tumor cell survival and adaptation. NRF2, in cooperation with SOX4, enhances metabolic reprogramming and drug resistance. NRF2 also upregulates key anti-ferroptotic genes, including SLC7A11, GPX4, and FSP1, leading to increased glutathione (GSH) synthesis and activation of the NADPH–CoQ antioxidant system. These processes collectively inhibit lipid peroxidation and ferroptosis, thereby facilitating tumor growth and therapy resistance. CoQ, coenzyme Q; FSP1, ferroptosis suppressor protein 1; GPX4, glutathione peroxidase 4; GSH, glutathione; HO-1, heme oxygenase-1; IL-6, interleukin-6; NF-κB, nuclear factor kappa B; NQO1, NAD(P)H quinone dehydrogenase 1; NRF2, nuclear factor erythroid 2-related factor 2; PPP, pentose phosphate pathway; SLC7A11, solute carrier family 7 member 11; SOX4, SRY-box transcription factor 4; STAT3, signal transducer and activator of transcription 3. suppressor protein 1; GSH, glutathione; CoQ, coenzyme Q; PPP, pentose phosphate pathway.

Taken together, NRF2-mediated treatment resistance is not driven by a single mechanism but rather establishes an adaptive survival network in HCC cells in response to therapeutic stress by coordinating multiple processes, including glutathione metabolism, NADPH supply, drug detoxification, drug efflux, and the maintenance of tumor-initiating cells. Although this sustained metabolic adaptation confers a significant survival advantage on tumor cells, it also makes them more dependent on NRF2-driven redox and metabolic networks, providing a theoretical foundation for the subsequent establishment of ferroptosis defense networks and the development of therapeutic strategies targeting metabolic vulnerabilities (**Figure 3**).

## 4 NRF2-mediated remodeling of the ferroptosis defense network

Ferroptosis is a form of programmed cell death driven by iron-dependent lipid peroxidation. A growing body of research indicates that ferroptosis is involved in the mechanisms of action of anticancer therapies such as sorafenib and is closely associated with the sensitivity of HCC to treatment (36, 75). In certain HCC models with sustained NRF2 activation, tumor cells can reduce treatment-induced lipid peroxidation levels by enhancing antioxidant defenses and regulating iron metabolism-related processes, thereby decreasing their sensitivity to ferroptosis (36, 84, 88, 90). Current research suggests that this anti-ferroptosis effect involves multiple interrelated regulatory pathways rather than relying on a single molecule. To systematically summarize existing research, this paper categorizes NRF2-mediated anti-ferroptosis regulation into three interrelated or potentially parallel defense mechanisms based on current pan-cancer knowledge and HCC-specific advances: ①the classical GSH–GPX4 system;② the GPX4-independent lipid radical scavenging system;③ the iron homeostasis regulatory system. These three-tiered defense networks collectively reduce sensitivity to ferroptosis by acting at three levels—lipid peroxide scavenging, lipid radical scavenging, and restriction of free iron accumulation—ultimately attenuating the cytotoxic effects induced by antitumor therapies. Currently, the first line of defense has been well supported by experimental studies in HCC; in contrast, some of the mechanisms underlying the latter two lines of defense still derive primarily from basic research or other tumor models, with relatively limited direct evidence in HCC; therefore, their specific roles require further validation (21, 24, 75, 91).

### 4.1 First line of defense: the classic GSH–GPX4 antilipid peroxidation system

The GSH–GPX4 axis is the most extensively studied component of the NRF2-mediated anti-ferroptosis network in HCC (36, 37, 75, 92). Persistently activated NRF2 directly binds to the antioxidant response element (ARE), induces SLC7A11 expression, promotes cysteine uptake, and simultaneously upregulates GCLC and GCLM, thereby enhancing glutathione (GSH) synthesis and continuously supplying reduced substrates to GPX4 (92–94). Subsequently, GPX4 uses GSH to reduce the cytotoxic phospholipid hydroperoxide (PL-OOH) to a lipid alcohol (PL-OH), thereby blocking the lipid peroxidation chain reaction and reducing susceptibility to ferroptosis (36, 75). Previous studies have shown that GSTZ1 deficiency leads to the accumulation of the metabolite succinylacetone, which inhibits NRF2 degradation by modifying key cysteine residues of KEAP1, thereby persistently activating the NRF2 signaling pathway and inducing the expression of SLC7A11 and GPX4, ultimately enhancing tumor cell resistance to sorafenib-induced ferroptosis; Restoring *GSTZ1* expression or inhibiting NRF2 can both reverse this resistance phenotype (37, 90). These results support the involvement of the GSH–GPX4 axis in sorafenib resistance-related processes, but its role in different HCC molecular subtypes requires further investigation.

### 4.2 Second line of defense: GPX4-independent lipid radical scavenging system

In addition to the classical GSH–GPX4 system, recent studies have revealed that cells also possess GPX4-independent lipid radical scavenging mechanisms. Among these, FSP1 is the representative molecule for which the most robust evidence currently exists (21). FSP1 is localized to the cell membrane and, by utilizing NAD(P)H, continuously reduces coenzyme Q10 (CoQ10) to ubiquinol. Ubiquinol can directly scavenge lipid peroxyl radicals and prevent the amplification of lipid peroxidation chain reactions, thereby forming a parallel anti-ferroptosis system independent of GPX4 (21, 95). In HCC, FSP1 is significantly overexpressed in human liver cancer tissues and is associated with poorer overall survival (OS) in patients; both genetic knockout and pharmacological inhibition of FSP1 (iFSP1) promote lipid peroxidation and ferroptosis, while inhibiting tumor growth and enhancing antitumor immunity in xenograft models, indicating that FSP1 is an important ferroptosis-inhibiting factor and a potential therapeutic target in HCC (96). Furthermore, studies on the molecular subtyping of liver cancer have shown that abnormal activation of NRF2/KEAP1 is often accompanied by the upregulation of antioxidant pathways such as FSP1, supporting the role of FSP1 as a key component of the NRF2 antioxidant network (97). However, evidence for direct NRF2-mediated transcriptional regulation of AIFM2/FSP1 currently derives primarily from non-HCC models such as lung cancer (24, 93), and validation of the direct mechanism in HCC remains limited. Therefore, existing evidence supports the involvement of the FSP1–CoQ10 system in GPX4-independent anti-ferroptosis regulation in HCC; however, whether it belongs to the direct downstream regulatory network of NRF2 requires further validation.

### 4.3 The third line of defense: regulation of iron homeostasis

In addition to clearing lipid peroxides, NRF2 can also reduce the underlying conditions for ferroptosis by regulating cellular iron homeostasis. Previous studies have shown that NRF2 can induce the expression of ferritin heavy chain (FTH1) and light chain (FTL), promoting ferritin formation, thereby reducing the intracellular labile iron pool (LIP) and decreasing Fenton reaction-mediated lipid peroxidation (98). Furthermore, NRF2 regulates the expression of the iron efflux protein ferroportin (FPN), thereby further contributing to the maintenance of cellular iron homeostasis (91, 99). On the other hand, the role of the classic NRF2 target gene HO-1 (HMOX1) in ferroptosis is distinctly context-dependent. Acute HO-1 activation can increase Fe²^+^ release and promote ferroptosis, whereas under chronic stress conditions, HO-1 may exert a cytoprotective effect by degrading pro-oxidative hemoglobin and generating antioxidant metabolites such as biliverdin and bilirubin. Therefore, the role of HO-1 in ferroptosis is highly context-dependent, and its specific effects may be influenced by factors such as cellular iron buffering capacity, redox status, and experimental models; a consensus has not yet been reached (75, 91, 99).

**Figure 4:**
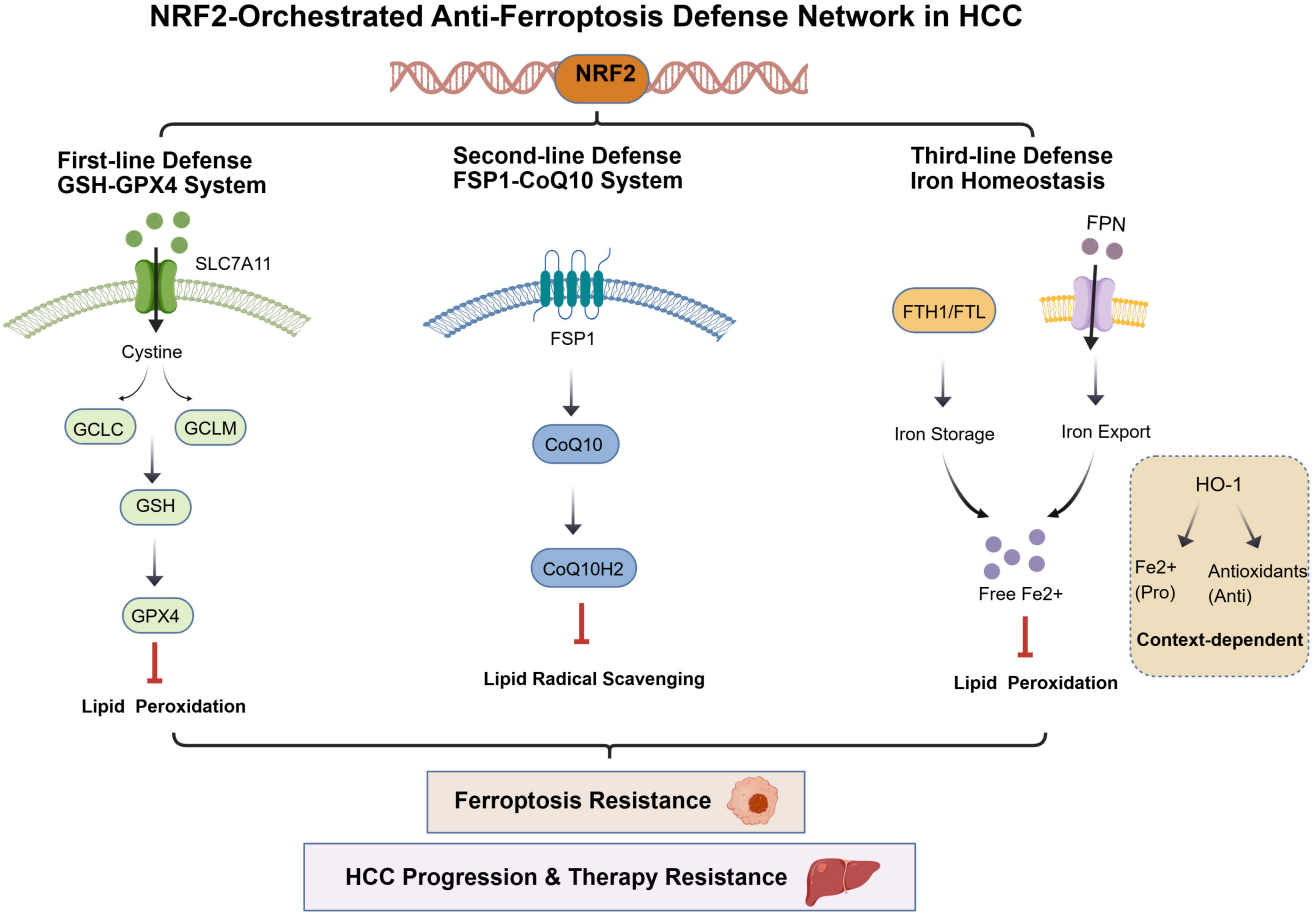
NRF2 orchestrates a three-layer anti-ferroptosis defense network in hepatocellular carcinoma. NRF2 coordinates three complementary anti-ferroptosis defense systems, including the GSH–GPX4 axis, the FSP1–CoQ10 axis, and iron homeostasis, to suppress lipid peroxidation and ferroptosis. These coordinated defense mechanisms promote hepatocellular carcinoma progression and therapy resistance. Notably, the role of HO-1 in ferroptosis is context-dependent and may exert either pro-ferroptotic or anti-ferroptotic effects. ARE, Antioxidant Response Element; CoQ10 / CoQ10H2, Coenzyme Q10 / Reduced Coenzyme Q10; FPN, Ferroportin; FSP1, Ferroptosis Suppressor Protein 1; FTH1 / FTL, Ferritin Heavy Chain 1 / Ferritin Light Chain; GCLC / GCLM, Glutamate-Cysteine Ligase Catalytic / Modifier Subunit; GPX4, Glutathione Peroxidase 4; GSH, Reduced Glutathione; HO-1, Heme Oxygenase 1; LIP, Labile Iron Pool; NRF2, Nuclear actor Erythroid 2-Related Factor 2; SLC7A11, Solute Carrier Family 7 Member 11.

Overall, most of the current evidence regarding NRF2’s regulation of iron homeostasis stems primarily from basic mechanism studies and other tumor models; direct functional validation in HCC remains relatively insufficient, and its specific contribution to HCC’s tolerance to ferroptosis requires further investigation (**Figure 4**).

## 5 Interaction of NRF2 with the tumor microenvironment

**Figure 5:**
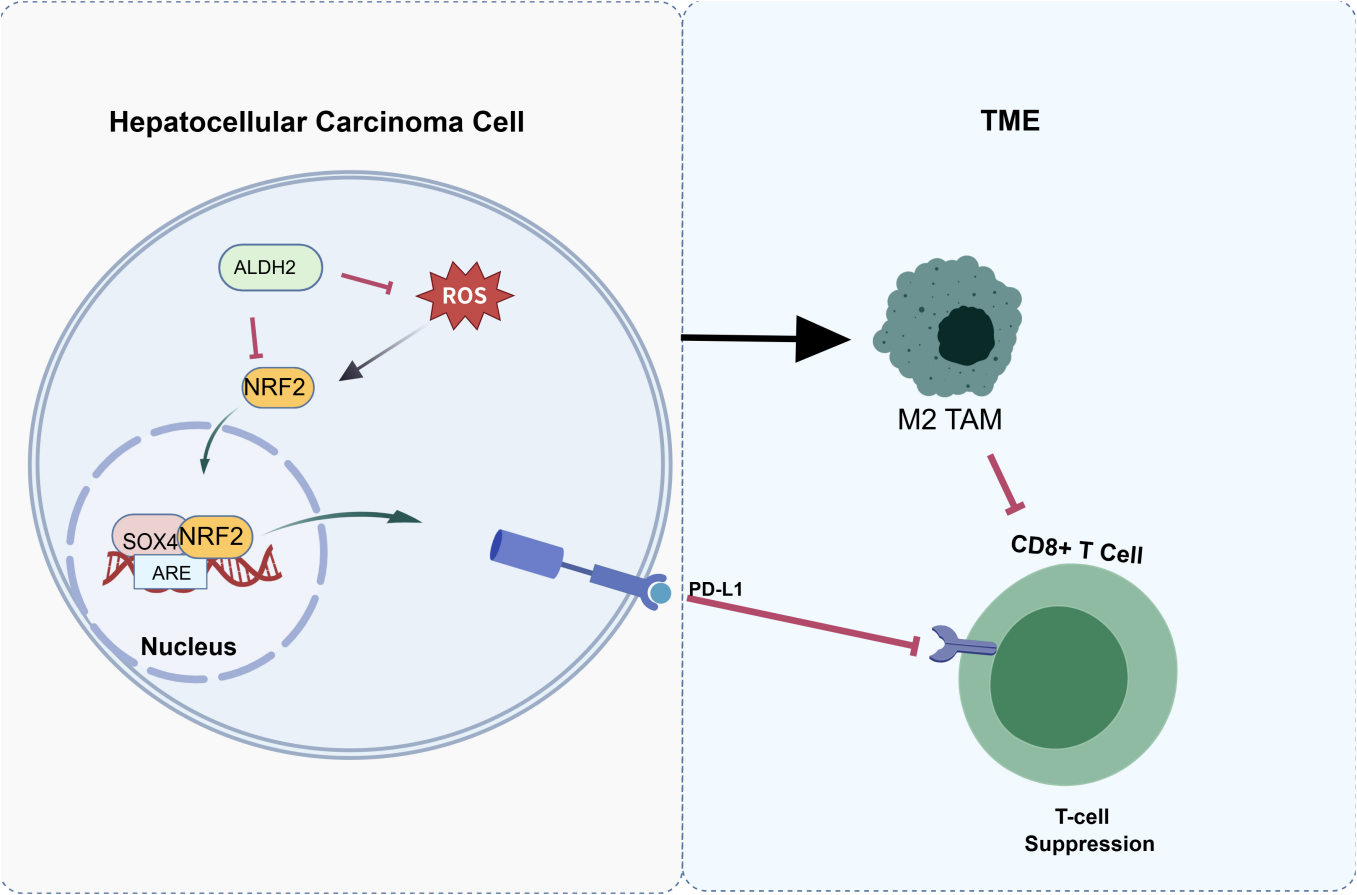
NRF2-mediated remodeling of the tumor microenvironment and immune evasion in hepatocellular carcinoma. In hepatocellular carcinoma (HCC) cells, NRF2 is activated by oxidative stress (ROS) and modulated by upstream regulators such as ALDH2. Activated NRF2 translocates into the nucleus, where it cooperates with SOX4 to bind antioxidant response elements (ARE) and promotes transcriptional programs that enhance serine metabolism and oxidative phosphorylation (OXPHOS) via PSPH. Concurrently, NRF2 upregulates PD-L1 expression and inhibits STING signaling, contributing to reduced innate immune activation. Within the tumor microenvironment (TME), NRF2-driven tumor cells promote polarization of tumor-associated macrophages toward an M2 phenotype (M2 TAM), which exerts immunosuppressive effects. These changes collectively suppress CD8^+^ T cell activity, leading to impaired anti-tumor immune responses and facilitating immune evasion. ALDH2, Aldehyde Dehydrogenase 2 Family Member; ARE, Antioxidant Response Element; CD8, Cluster of Differentiation 8; M2 TAM, M2-polarized Tumor-Associated Macrophage; NRF2, Nuclear Factor Erythroid 2-Related Factor 2; OXPHOS, Oxidative Phosphorylation; PD-L1, Programmed Death-Ligand 1; PSPH, Phosphoserine Phosphatase; ROS, Reactive Oxygen Species; SOX4, SRY-Box Transcription Factor 4; STING, Stimulator of Interferon Genes; TME, Tumor Microenvironment.

In addition to regulating the redox homeostasis within tumor cells, NRF2 also influences the immunological state of the tumor microenvironment through metabolic remodeling. Existing studies indicate that NRF2 activation is associated with the polarization of tumor-associated macrophages toward an M2-like phenotype, the maintenance of tumor stemness, and treatment resistance, and may also influence CD8^+^ T cell-mediated antitumor immune responses (80, 100). Furthermore, ALDH2 downregulates PD-L1 expression and enhances antitumor immune responses by inhibiting ROS/NRF2-mediated autophagy activation, suggesting that NRF2 may be involved in regulating immune checkpoint-related signaling (101). Taken together, NRF2-mediated metabolic regulation may not only help tumor cells adapt to oxidative stress environments but may also influence immune processes associated with the tumor microenvironment (80, 100, 101). However, most of the current evidence for these mechanisms stems from studies on other solid tumors, such as lung and pancreatic cancer; direct evidence in HCC remains relatively limited, and the specific regulatory mechanisms and clinical significance still require further investigation (102, 103) (**Figure 5**).

## 6 NRF2-driven metabolic vulnerability: a new approach to HCC treatment

### 6.1 Theoretical basis of metabolic vulnerability

In recent years, metabolic vulnerability has gradually emerged as a key theoretical framework in cancer metabolism research. Extensive studies have shown that tumor cells coordinate core metabolic pathways—including those for carbohydrates, lipids, and amino acids—through metabolic reprogramming to meet biological demands such as sustained proliferation, biosynthesis, and maintenance of redox homeostasis, while simultaneously enhancing their adaptability to nutrient deprivation, oxidative stress, and the complex tumor microenvironment (104–106). Furthermore, cancer cells are capable of utilizing different nutritional substrates (such as glucose and glutamine) and various nutrient acquisition mechanisms to maintain energy supply, biomass synthesis, and redox balance, thereby supporting their sustained growth and survival (105, 106).

Based on this, a growing body of research suggests that the long-term maintenance of an adaptive metabolic state in tumor cells is often accompanied by increased metabolic dependency on certain key metabolic pathways, nutrient substrates, or redox regulatory networks. When these key metabolic processes are targeted by pharmacological or genetic interventions, some tumor cells—due to their limited compensatory capacity—are more susceptible to growth inhibition or cell death; in contrast, normal tissues typically possess greater metabolic regulatory capacity and thus exhibit relatively better tolerance to the same interventions (104–106). This characteristic forms the theoretical foundation of metabolic vulnerability and provides an important theoretical basis for metabolite-targeted therapy.

In hepatocellular carcinoma (HCC), persistently activated NRF2 regulates multiple metabolic pathways, including glutathione (GSH) metabolism, NADPH production, and serine metabolism, thereby helping cells maintain redox homeostasis and enhancing their ability to cope with stressful environments (84, 104). Therefore, the NRF2-mediated metabolic network may lead HCC cells to develop a certain degree of metabolic dependence during long-term adaptation, providing a theoretical basis for subsequent therapeutic interventions that exploit metabolic vulnerability.

### 6.2 NRF2-driven metabolic reprogramming confers metabolic advantages in HCC

Persistently activated NRF2 is not only a classic antioxidant transcription factor but also a key regulator involved in cellular metabolic reprogramming. Previous studies have shown that in HCC, NRF2 activation promotes the remodeling of metabolic pathways such as those involving glucose and serine, thereby enhancing the ability of tumor cells to adapt to oxidative stress and sustain proliferation (76, 80). Recent studies have further demonstrated that NRF2 not only enhances PPP and TCA cycle activity but also induces tumor cells to become dependent on the metabolic networks involving the PPP, NADPH, and GSH (107–110). In HCC, abnormal NRF2 activation is also closely associated with glucose metabolism reprogramming and facilitates tumor cell adaptation to a persistent oxidative stress environment (76, 111). However, current evidence that NRF2 directly regulates key PPP enzymes such as G6PD, PGD, and TKT primarily comes from studies of other tumors and basic mechanisms; its specific regulatory mechanisms in HCC remain to be further elucidated. Furthermore, NRF2 can induce the expression of antioxidant-related genes such as SLC7A11, GCLC, and GCLM, thereby promoting glutathione (GSH) synthesis and maintaining cellular redox homeostasis; this process is closely associated with HCC cells’ adaptation to oxidative stress and resistance to ferroptosis (16, 112). Notably, NRF2 promotes the PHGDH-mediated serine synthesis pathway (SSP), enhancing serine metabolism in HCC cells and sustaining tumor growth (108). PHGDH has also been identified as a key metabolic factor driving sorafenib resistance, further suggesting that serine metabolism is involved in the metabolic adaptation process of HCC (114). On the other hand, basic mechanistic studies have suggested that NRF2 may also promote glutamine metabolism and provide metabolic substrates for the tricarboxylic acid cycle, one-carbon metabolism, and NADPH production; however, direct evidence for this mechanism in HCC remains relatively limited and requires further validation (115). Furthermore, existing studies suggest that NRF2-mediated metabolic reprogramming may regulate epigenetic processes—such as histone acetylation—by influencing the levels of key metabolic intermediates, such as acetyl-CoA, thereby affecting the transcriptional expression of HCC-related genes (76, 115). Overall, existing studies indicate that NRF2 participates in coordinating energy metabolism, biosynthesis, and redox regulation, helping HCC cells adapt to oxidative stress, nutritional restriction, and therapeutic stress (76, 80, 111, 113). These metabolic changes may further increase tumor cells’ dependence on certain metabolic networks, providing a theoretical basis for subsequent exploration of metabolic vulnerability.

### 6.3 From metabolic advantage to dependency: the evolution of metabolic vulnerability

Although sustained metabolic reprogramming endows HCC cells with the ability to adapt to oxidative stress and therapeutic pressures, long-term maintenance of this metabolic state may also increase their dependence on certain key metabolic pathways. Therefore, the metabolic advantage conferred by sustained NRF2 activation may further increase HCC cells’ dependence on the maintenance of redox homeostasis, glutathione metabolism, and certain antioxidant metabolic networks, thereby creating potential metabolic vulnerability (116–118). When NRF2-related metabolic networks are disrupted by pharmacological or genetic interventions, the redox homeostasis of HCC cells can be disrupted, accompanied by increased lipid peroxidation and heightened sensitivity to ferroptosis (117, 118). For example, silencing glutamate-oxaloacetate transaminase 2 (GOT2) can reshape glutamine metabolism and enhance the sensitivity of HCC cells to glutamine synthetase (GLS) inhibitors, suggesting the existence of a metabolically dependent target in HCC (119). Furthermore, reducing NAT10-mediated NRF2 mRNA stability decreases NADPH and GSH levels, while inhibiting the MCM4–NRF2 signaling pathway promotes sorafenib-induced ferroptosis, further supporting the notion that targeting NRF2-related metabolic networks may enhance therapeutic sensitivity in HCC (117, 118). In addition to redox metabolism, NRF2 can also enhance the resistance of HCC cells to lipid peroxidation and ferroptosis by regulating the SLC7A11/GPX4 antioxidant system and molecules related to iron homeostasis (20, 118). Therefore, these key metabolic processes involved in maintaining the metabolic adaptation of tumor cells may serve as potential targets for metabolic interventions worthy of further investigation. Overall, existing studies suggest that while NRF2-mediated metabolic reprogramming endows HCC cells with metabolic adaptability, it may also increase their dependence on certain antioxidant and metabolic networks, thereby creating potential metabolic vulnerabilities and providing a theoretical basis for subsequent metabolically targeted therapeutic strategies.

### 6.4 Leveraging metabolic vulnerabilities: NRF2-targeted combination strategies

Based on the potential metabolic vulnerabilities arising from NRF2-driven metabolic dependence, combined interventions targeting its antioxidant metabolic networks have become an important research direction in recent years for enhancing HCC treatment sensitivity. A growing body of evidence indicates that NRF2-mediated metabolic reprogramming is involved in the development of acquired resistance to molecularly targeted therapies such as sorafenib in HCC. During sorafenib treatment, HCC cells can activate stress-regulatory networks such as p62/KEAP1/NRF2, NRF2/HO-1/GPX4, and ABCC5/NRF2, accompanied by the upregulation of antioxidant-related molecules such as GPX4 and SLC7A11, thereby enhancing their ability to maintain redox homeostasis and their tolerance to ferroptosis (112, 120–123). Consequently, both pharmacological inhibition and gene silencing of NRF2 can increase the sensitivity of HCC cells to sorafenib (114, 123). These studies suggest that although NRF2-mediated antioxidant and metabolic adaptations promote treatment resistance, they may also render tumor cells more dependent on related redox defense networks, providing a theoretical basis for combination therapies targeting these metabolic dependencies. Furthermore, various combination intervention strategies targeting NRF2 signaling have demonstrated potential in HCC models. For example, nutritional restriction can promote ferroptosis and enhance sorafenib efficacy by regulating the NRF2/HO-1/GPX4 signaling pathway; the natural IGF1R inhibitor picropodophyllin can block the AKT/NRF2/SLC7A11 and AKT/NRF2/SLC40A1 signaling pathways, thereby increasing sensitivity to ferroptosis; curcumin, in turn, can regulate the p62–KEAP1–NRF2 signaling pathway to promote lipid peroxidation and ferroptosis (47, 107, 122) (**Table 1**).

**Table 1:** Summary of NRF2-mediated metabolic advantages, dependencies, and intervention strategies.

Mechanistic dimension	Metabolic advantages	Metabolic vulnerabilities	Potential intervention strategies
Carbohydrate Metabolism	Upregulates key enzymes of the PPP (G6PD, TKT, PGD), enhancing NADPH production.	Redox addiction: Highly dependent on NADPH produced by the PPP to maintain redox homeostasis.	G6PD inhibitors(polydatin) (10, 52, 53, 99, 108, 114)
Amino Acid Metabolism	Activates glutamine breakdown and upregulates transporters and metabolic enzymes to support biosynthesis.	Glutamine addiction: Large amounts of glutamine are redirected to GSH synthesis and nucleotide metabolism.	GLS1 inhibitor (CB-839) (10, 11, 13, 91, 115, 116)
Resistance to ferroptosis	Activates the SLC7A11-GSH-GPX4 axis; reduces the free iron pool via FTH1/FPN.	Defense rigidity: Highly dependent on GSH levels and iron homeostasis to evade lipid peroxidation.	Ferroptosis inducers (erastin, RSL3, sorafenib) (9, 41, 42, 71, 78, 108)
Epigenetics	Maintains Acetyl-CoA levels and ensures transcription of target genes through histone acetylation.	Transcriptional threshold vulnerability: Limited metabolite supply blocks gene expression at the epigenetic level	Epigenetic modulators (HDAC inhibitors), and metabolic–epigenetic co-targeting strategies (8, 52, 92, 97, 100).
Homeostasis Maintenance	Stabilizes mRNA via NAT10; inhibits protein degradation using p62 or IGF2BP3.	Factor Dependency: High expression of NRF2 is highly dependent on specific RNA processing or protein interactions.	NRF2 Inhibitors (brusatol, ML385) (22, 43, 44, 70, 75, 97, 100)

NRF2, nuclear factor erythroid 2-related factor 2; PPP, pentose phosphate pathway; G6PD, glucose-6-phosphate dehydrogenase; TKT, transketolase; PGD, 6-phosphogluconate dehydrogenase; NADPH, nicotinamide adenine dinucleotide phosphate (reduced form); GLS1, glutaminase 1; GSH, glutathione; SLC7A11, solute carrier family 7 member 11; GPX4, glutathione peroxidase 4; FTH1, ferritin heavy chain 1; FPN, ferroportin; HDAC, histone deacetylase; m6A, N6-methyladenosine; NAT10, N-acetyltransferase 10; p62, sequestosome 1 (SQSTM1); IGF2BP3, insulin-like growth factor 2 mRNA-binding protein 3. Polydatin, a natural G6PD inhibitor; CB-839 (telaglenastat), a selective GLS1 inhibitor; Erastin, a system Xc^−^; inhibitor and ferroptosis inducer; RSL3, a direct GPX4 inhibitor; Sorafenib, a multikinase inhibitor with ferroptosis-inducing activity in HCC; Brusatol, an experimental NRF2 inhibitor; ML385, an inhibitor of NRF2 transcriptional activity.

## 7 Controversies, unresolved issues, and future prospects

Although significant progress has been made in recent years in the study of NRF2 in hepatocellular carcinoma (HCC), its roles in the regulation of oxidative stress, metabolic reprogramming, treatment resistance, and ferroptosis have been supported by a large body of experimental research (61, 124, 125). However, the maturity of research evidence varies across different mechanisms; some regulatory networks are still primarily derived from basic research, individual studies, or other tumor models, and their biological significance and clinical relevance in HCC require further validation. Overall, several issues warrant attention in current research. First, NRF2 exhibits marked context-dependence, and its biological functions are influenced by various factors, including tissue type, disease stage, and mode of activation (9, 16, 69). It is currently widely accepted that NRF2 helps maintain redox homeostasis and exerts a cytoprotective effect in normal tissues, whereas in tumors with sustained abnormal activation, it may promote the adaptation of tumor cells to an oxidative stress environment (67, 126). However, the molecular basis for the shift of NRF2 from a protective to a tumor-promoting role has not yet been fully elucidated; conclusions drawn from different studies also vary to some extent, and the specific regulatory mechanisms require further investigation.

In recent years, the NRF2-related regulatory network has continued to expand, but there are marked differences in the strength of evidence supporting different mechanisms. Currently, the classical KEAP1–NRF2 regulatory axis, p62-mediated non-classical activation, and the GSH–GPX4 antioxidant system have been well supported by HCC research (26, 27, 36, 48, 55, 124, 125, 127). In contrast, the FSP1–CoQ10 system, certain immune regulatory mechanisms, glutamine metabolism, and some epigenetic regulatory pathways are currently inferred primarily from other tumor models or limited HCC studies, and direct evidence for their roles in HCC remains relatively scarce (16, 18, 21, 24, 93, 128). Furthermore, the SOX4–NRF2 regulatory axis, IGF2BP3-related positive feedback, and recently reported regulatory mechanisms such as NAT10 and MCM4 are currently derived mostly from single studies and still lack validation through independent studies and clinical samples (60, 80, 117, 118). Therefore, when interpreting the above research findings, care should be taken to distinguish between direct evidence from HCC studies and extrapolated evidence from studies on other cancer types, and a comprehensive evaluation should be conducted based on the strength of the evidence.

In addition, the theory of metabolic fragility, proposed in recent years, has provided a new research perspective for understanding NRF2-mediated metabolic adaptation (72, 73). Previous studies have suggested that sustained activation of NRF2 may increase the dependence of HCC cells on the maintenance of redox homeostasis, glutathione metabolism, and certain antioxidant networks. When these metabolic processes are disrupted by pharmacological or genetic interventions, increased sensitivity to ferroptosis or improved treatment responses have been observed in some cellular and animal experiments (68, 69). However, current research remains largely at the basic experimental stage. There is still insufficient evidence to support whether stable and reproducible metabolic dependencies exist across HCC cases with different etiologies and molecular subtypes, or whether these findings can be translated into clinical treatment strategies (36). Furthermore, NRF2 also plays a crucial cytoprotective role in normal liver tissue; therefore, when conducting NRF2-related interventions in the future, it will be necessary to further evaluate their potential impact on the redox homeostasis of normal tissue and optimize therapeutic selectivity to enhance the feasibility of clinical application (36, 48).

Overall, current research on NRF2 in HCC has gradually expanded from classical redox regulation to multiple areas, including metabolic reprogramming, ferroptosis, and the tumor microenvironment; however, the maturity of evidence for different mechanisms still varies significantly, and some research conclusions require further validation through HCC-specific studies (73, 74, 76). Future research should combine research strategies such as large-scale clinical cohorts, single-cell sequencing, spatial transcriptomics, integrated multi-omics analysis, and patient-derived organoids to systematically validate NRF2-related regulatory networks and further clarify the patterns of NRF2 activation and metabolic heterogeneity in HCC with different etiologies and molecular subtypes (14–16, 18). At the same time, a reliable system of NRF2-related biomarkers should be established to evaluate their clinical utility in patient stratification, treatment response prediction, and combination therapy, thereby providing a more robust theoretical basis for precision treatment of HCC (74, 89).

## 8 Conclusion

Overall, NRF2 exhibits marked context- and stage-dependence in HCC, with its function gradually shifting from maintaining tissue redox homeostasis in the early stages to supporting tumor cell metabolic adaptation, ferroptosis resistance, and treatment resistance. A growing body of evidence suggests that while sustained activation of NRF2 confers a metabolic advantage to HCC cells, it simultaneously fosters a persistent dependence on networks regulating redox homeostasis, glutathione metabolism, serine metabolism, and ferroptosis defense, thereby creating metabolic vulnerabilities that can be targeted by therapeutic interventions. Future efforts should focus on further investigating HCC-specific mechanisms, establishing a reliable system of NRF2-related biomarkers, and developing precision treatment strategies based on metabolic dependence, ferroptosis, and combined immunotherapeutic interventions to advance the translation of NRF2-related basic research into clinical practice.
