# Supplementary material for: The NRF2 signaling pathway in hepatocellular carcinoma: dual roles, epigenetic reprogramming, and therapeutic opportunities in metabolic vulnerability
Source: Front Oncol. 2026 Aug 5;16:1874956. doi: 10.3389/fonc.2026.1874956 (PMC13487741; doi:10.3389/fonc.2026.1874956)
Supplement: Supplementary file 1 [file Table1.docx]

Table 1. NRF2-mediated metabolic reprogramming, potential metabolic dependencies and therapeutic opportunities in hepatocellular carcinoma

| Mechanistic category | NRF2-mediated metabolic reprogramming | Potential metabolic dependency | Potential therapeutic strategy |
| --- | --- | --- | --- |
| Glucose metabolism | Promotes glucose flux into the pentose phosphate pathway (PPP), increases NADPH production, and supports redox homeostasis and biosynthesis | Increased dependence on PPP activity and NADPH supply | Targeting glucose metabolism, PPP or redox metabolic networks (18, 108-110) |
| Amino acid metabolism | Reprograms glutamine metabolism and PHGDH-mediated serine biosynthesis to sustain antioxidant metabolism and proliferation | Increased dependence on glutamine metabolism and serine biosynthesis | Targeting GLS, PHGDH and related metabolic enzymes (80, 113, 114) |
| Ferroptosis defense | Activates the SLC7A11–GSH–GPX4 antioxidant system and enhances ferroptosis resistance | Increased reliance on GSH homeostasis and GPX4-mediated antioxidant defense | Targeting the SLC7A11–GPX4 axis combined with ferroptosis induction (21, 107, 110) |
| Redox adaptation | Maintains NRF2-driven antioxidant metabolism to improve oxidative stress adaptation | Increased dependence on NRF2-mediated redox regulation | Combination strategies targeting NRF2-associated antioxidant pathways (16, 19, 20) |
| Upstream regulation of NRF2 | Persistent NRF2 activation mediated by KEAP1 mutation, p62-dependent noncanonical activation, NAT10 and MCM4 | Dependence on sustained NRF2 activation | Targeting upstream regulatory pathways of NRF2 (13, 17, 117, 118) |
